# Improving Adherence to the Lead Exposure Protocol at Boston Medical Center’s Pediatric Clinic

**DOI:** 10.1097/pq9.0000000000000793

**Published:** 2025-02-05

**Authors:** Julie R. Barzilay, Anthony J. Mell, MaryKate Driscoll, Priscilla Gonzalez, Sarah Meyers, Noah Buncher

**Affiliations:** From the * Boston Medical Center, Boston, Mass.; † Boston Children’s Hospital, Boston, Mass; ‡ Monroe Carell Jr. Children’s Hospital at Vanderbilt; § Children’s Hospital of Philadelphia.

## Abstract

**Introduction:**

Using plan-do-study-act cycles, our team aimed to increase mean provider adherence to the Lead Exposure Protocol at the Boston Medical Center Pediatric Primary Care Clinic from 16% (baseline global mean provider adherence) to 80% from April 1, 2021, to February 1, 2023, thereby curbing the secondary effects of lead exposure.

**Methods:**

Our team performed a chart review of patients 6 months to 5 years of age with blood lead levels (BLLs) ≥2 µg/dL (n = 853) to track provider adherence to Boston Medical Center’s Lead Exposure Protocol. We created p charts to track the efficacy of interventions to improve adherence. Interventions included (1) electronic medical record SmartPhrases, (2) provider education, (3) provider feedback, (4) implementation of a follow-up nursing workflow, and (5) simplification of nursing workflow.

**Results:**

For BLL 2–4 µg/dL (n = 783), a centerline shift in provider adherence was observed, with >8 points above the preintervention mean after intervention (2) and an increase in mean adherence from 14.1% to 50%. For BLL 5–9 µg/dL (n = 58), no centerline shift was observed, with only 6 points above the upper control limit after intervention (4). The 2–4 µg/dL range changes indicate special cause variance and system change. Global mean provider adherence increased by 3.3 times to 53%.

**Conclusions:**

Simple, low-cost process changes improved adherence to complex guidelines for managing lead-exposed children in the primary care setting. Similar interventions could be implemented on a broader scale to standardize the management of other routine pediatric screens.

## INTRODUCTION

Elevated blood lead levels (BLLs) are associated with adverse developmental and behavioral outcomes.^1^ Lead exposure is associated with hearing and language delays, brain and nervous system damage, decreased intelligence quotient, and slowed growth and development.^2^ Symptomatically, it can cause abdominal pain, constipation, depression, irritability, nausea, and distraction.^3^ No safe BLL threshold has been identified for children.^4^

Childhood lead exposure remains a significant public health burden despite improvements in federal and state regulations—particularly in light of the COVID-19 pandemic when hundreds of thousands of children missed their screenings and children spent more time indoors.^5–7^ The most significant source of lead exposure nationwide and in Massachusetts is paint dust. In 2017, 88% of lead poisoning cases (lead poisoning as defined by the state of Massachusetts is any BLL ≥10 µg/dL for a child younger than 6 years of age) were due to exposure to lead paint.^8^ Homes built before 1978 likely contain lead paint, which can chip and turn into dust or contaminate soil around homes. Massachusetts has the fourth oldest housing stock in the country, with approximately 70% of houses built before 1978.^6^ Alternative, though less common, sources of lead exposure include spices, herbal remedies, imported toys and cookware, or water contaminated by lead pipes.^9^

The lead level defined by the Centers for Disease Control and Prevention as the threshold for intervention, which had been 5 µg/dL since 2012, was changed to 3.5 µg/dL in October 2021. This value is based on the 97.5 percentile of the blood lead distribution in US children 1–5 years of age.^10^ In the past, much higher cutoffs were used. Revising the reference value means roughly double the number of screened children require follow-up.^4^ Even before this change, provider interventions for elevated BLL have been inconsistent, making this a ripe area for quality improvement (QI) efforts.^11^

The Massachusetts Department of Public Health recommends screening children’s BLLs at ages 9–12 months, 2 and 3 years, and again at 4 years if they live in a high-risk area.^12^ This study aimed to measure and improve adherence to correct follow-up steps per clinic protocol when a child screens positive for lead exposure. The Boston Medical Center Elevated Blood Lead Level Treatment Protocol (BMC Lead Exposure Protocol) was developed based on 3 evidence-based guidelines: those of the American Academy of Pediatrics, the Centers for Disease Control and Prevention, and the Massachusetts Department of Public Health.^13–15^

The primary goal of this study was to improve guideline-adherent care, thereby curbing or halting the secondary effects of lead exposure. Using rapid plan-do-study-act cycles, we aimed to increase baseline mean provider adherence to the Lead Exposure Protocol at the BMC Pediatric Primary Care Clinic from 16% to 80% between April 1, 2021, and February 1, 2023.

## METHODS

### Setting

The BMC Pediatric Primary Care Clinic is a large, urban academic center with 25 providers serving approximately 14,000 patients. It includes a resident clinic with 30–35 resident providers supervised by preceptors. The patients served by BMC are largely from medically underserved neighborhoods in the Boston area and include many new immigrant patients. Most of the BLL follow-ups in the clinic occur by telephone. Providers at BMC utilize a Health Insurance Portability and Accountability Act of 1996-compliant phone interpreter service to call patients for clinical updates. In the clinic setting, in-person interpreters and iPad interpreters are also available. In a large clinic with rotating trainees, it can be challenging to disseminate new information and protocols—especially complex ones like the Lead Exposure Protocol. However, there is precedent in the literature: in a 2019 study from Cincinnati Children’s Hospital by Brown et al,^16^ a similarly urban, outpatient academic practice aimed to standardize clinical responses to lead results and described interventions that proved effective in improving adherence to lead guidelines. Using that study as a model, our team adopted several of the interventions to our practice setting and introduced novel interventions.^16^ The team comprised 4 pediatric residents in the Boston Combined Residency Program and 1 attending primary care physician at BMC, who was also the Medical Director of the BMC Lead Clinic during the data collection and some of the data analysis period of the study. The BMC Lead Clinic is a specialty clinic offering clinical, environmental, and educational services to children with BLLs ≥10 µg/dL. During the data collection and analysis period, all of the residents graduated and became attending physicians or clinical fellows in pediatric subspecialties.

### Data Collection

**Fig. 1.**
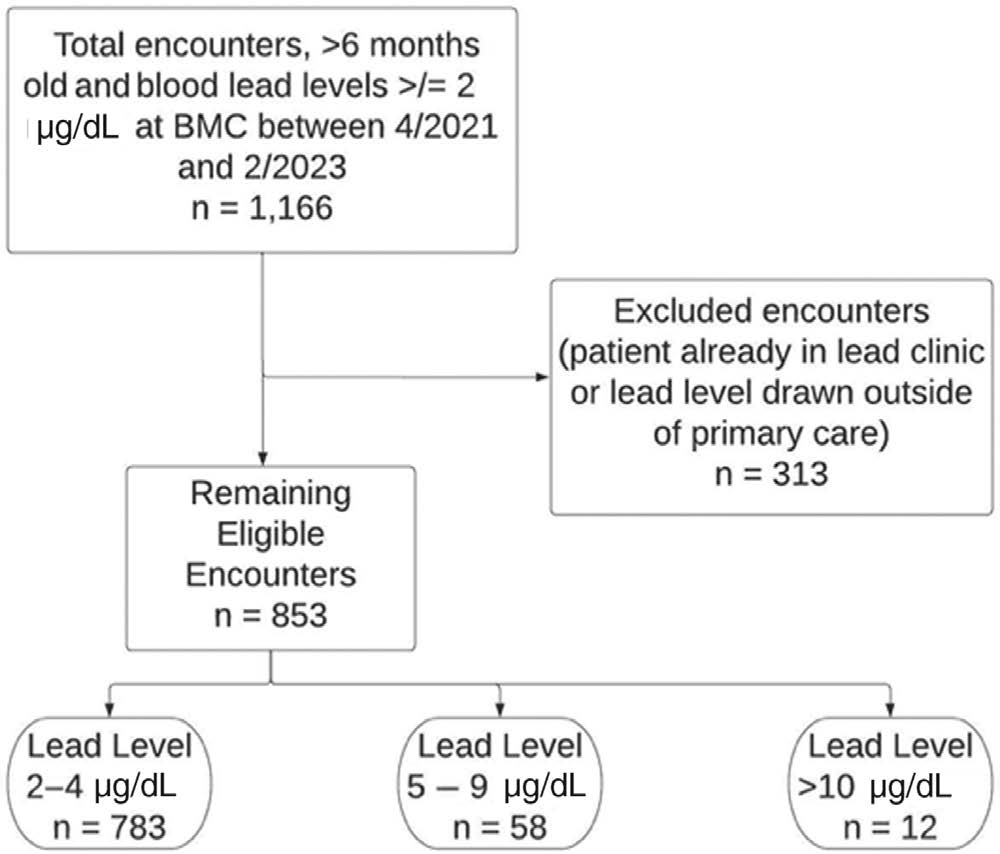
Flow diagram depicting inclusion and exclusion of charts for review and breakdown by BLL during the study period, April 1, 2021, to February 1, 2023.

Our project was reviewed by the institutional review board (IRB). It was deemed exempt for review of electronic medical record (EMR) charts for patients 6 months to 5 years of age with an elevated BLL greater than or equal to 2 µg/dL at the BMC Pediatric Primary Care Clinic (Fig. 1). Only patient encounters that represented a first abnormal BLL result were included in the study—follow-up values from subsequent encounters were excluded. The BMC Clinical Data Warehouse for Research (CDW-R) shared Medical Record Numbers for patients meeting these criteria each month, paired with a matching random de-identified numerical assignment and key for data analysis. Each researcher was randomly assigned a set of charts to review. A binary “yes” or “no” question-based system was used to record whether each step in the protocol had been followed for the respective BLL. If a patient was already followed in the BMC Lead Clinic , or if the lead level was obtained by a specialty clinic or the Emergency Department, that chart was excluded.

Data were collected over 6 months to establish a baseline mean provider adherence rate, calculated by taking the mean provider adherence values for the first 6 months of the study. The baseline mean provider adherence level was 16% for all lead levels combined, 14.1% for the 2–4 µg/dL range, and 44% for the 5–9 µg/dL range. The baseline mean provider adherence level for lead levels ≥10 µg/dL was 0%; however, there were too few encounters (n = 12 over the course of the study) to meaningfully track this range with a p chart or assess change. After establishing baseline mean provider adherence, our team began implementing interventions and engaging in plan-do-study-act cycles to evaluate their effectiveness.

### Outcome Measure

**Fig. 2.**
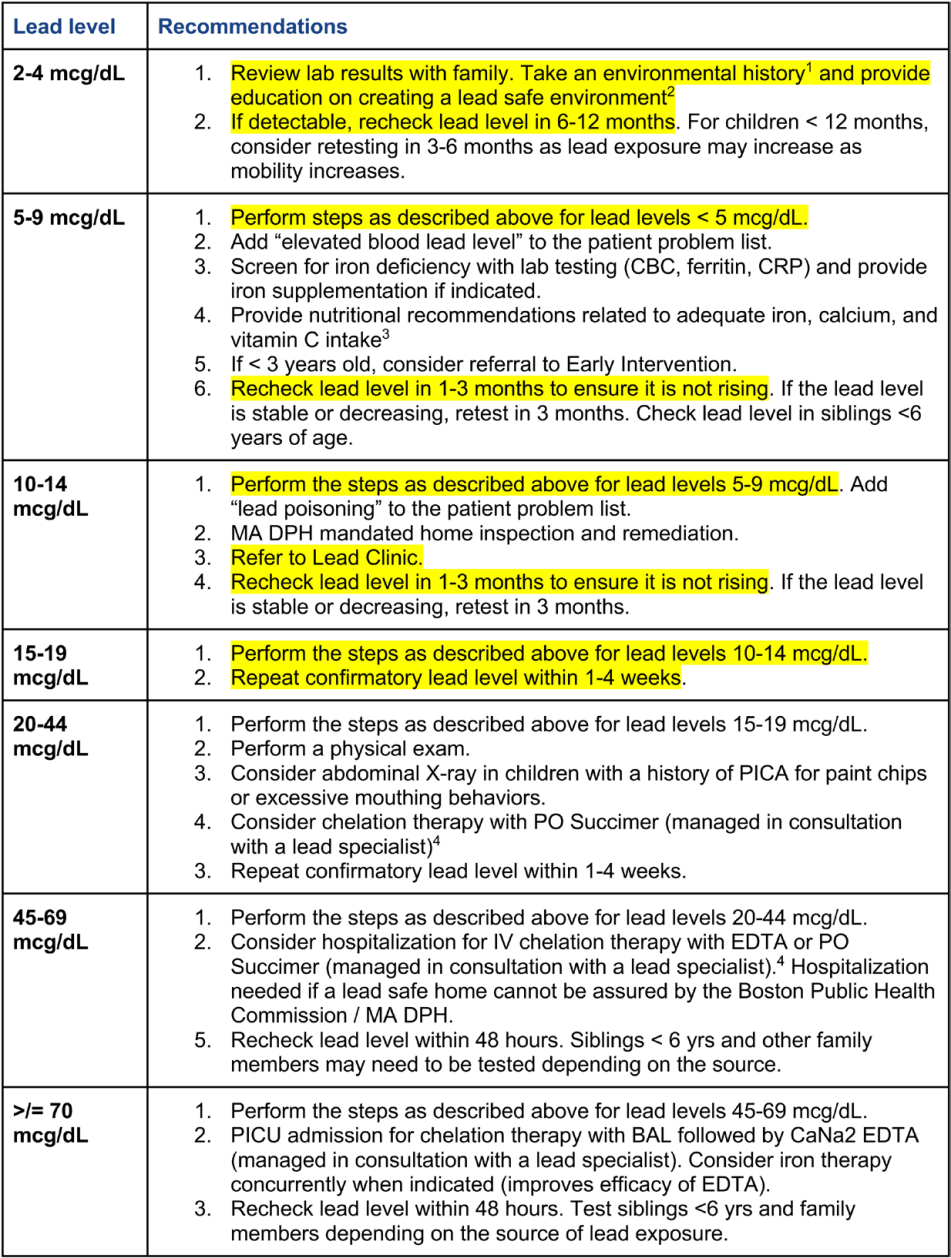
BMC elevated BLL treatment protocol (“lead exposure protocol”), critical protocol steps highlighted.

The primary outcome measure was the percent provider adherence to the protocol. The percentage of providers adhering to “critical” steps in the protocol for each BLL range each month was tracked. The critical steps are a subset of the recommended steps that constitute a minimum of care required. Providers were not made aware of this distinction. Figure 2 shows the full Lead Exposure Protocol, highlighting the critical steps. Adherence to all critical steps for a given patient was required for an encounter by that provider to be counted as adherent.

### Interventions

The COM-B model (Capability, Opportunity, Motivation-Behavior) drives several interventions. This model states that three factors must be present for behavior to change: capability, opportunity, and motivation.^17^ Each intervention aimed to improve at least one of these tenets.

**Fig. 3.**
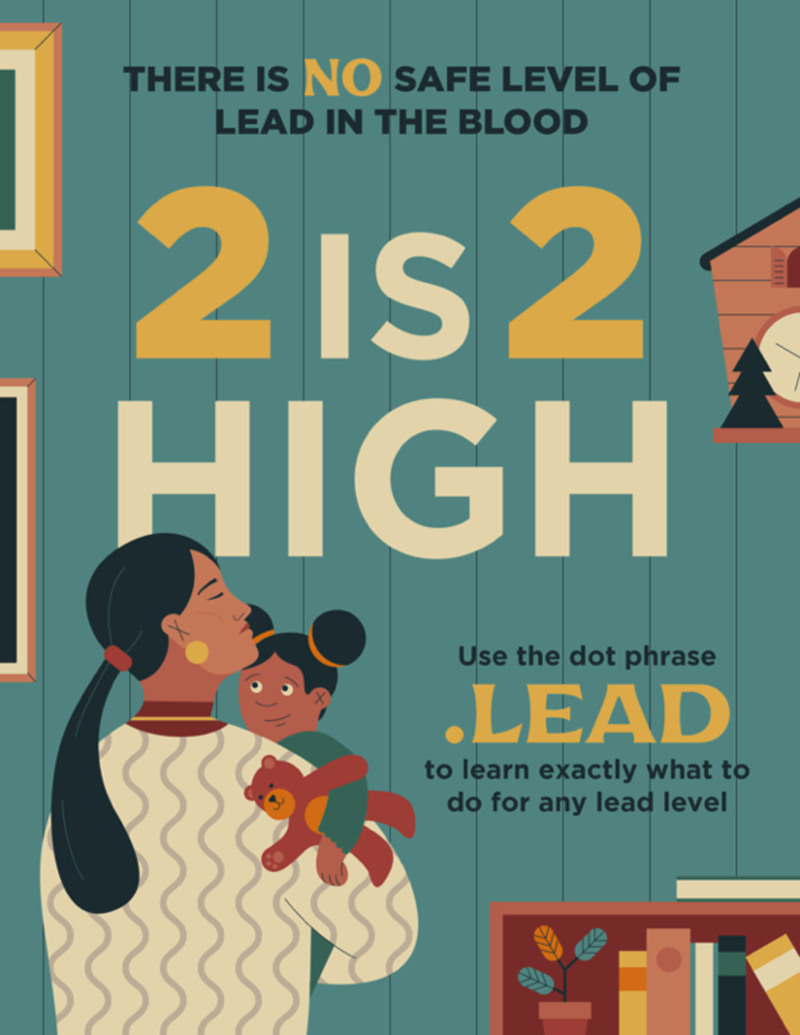
“2 is 2 High” educational campaign graphic created by Dr. Ryan Brewster, MD.

The following is a description of each intervention (**see Table 1, Supplemental Digital Content,** http://links.lww.com/PQ9/A635):

1. SmartPhrase introductions (September 2021) (COM-B Tenet: capability): EMR SmartPhrases (**see Document 2, Supplemental Digital Content,** http://links.lww.com/PQ9/A636) were created for each lead level range, which contained protocol-adherent follow-up steps as well as informational materials to share with families. SmartPhrases were shared with providers via email, announcements at monthly provider meetings, stickers on computers, and word-of-mouth.
2. Resident and attending educational/promotional campaign (September–October 2021) (COM-B Tenet: motivation): Our team presented didactic sessions about lead screening, exposure, and management for residents and attending providers. The team also launched an awareness campaign featuring stickers, posters, magnets, and buttons with a graphic designed by Dr. Ryan Brewster (Fig. 3). The emphasis was that “2 is 2 High,” meaning there is no safe BLL and that a BLL of 2 µg/dL is actionable per the protocol.
3. Directed feedback to attending providers (April 2022) (COM-B Tenet: opportunity): Our team sent monthly EMR messages in the style of academic detailing to providers with low adherence levels, sharing the protocol and SmartPhrases.
4. Dedicated pathway for trained nurses to follow-up on lead results (May 2022) (COM-B Tenet: capability): We trained all clinic nurses on the Lead Exposure Protocol. Our team publicized that providers could forward BLL results in the 2–4 µg/dL range to the nursing pool. Nurses followed up on results and documented steps taken. Providers were responsible for following up on BLLs above this range.
5. Streamlining of nurse follow-up pathway (November 2022) (COM-B Tenet: motivation): Responding to provider feedback, our team clarified and simplified how providers could forward results to nursing pools. We assigned individual nurses to groups of providers (who were separated into teams with different color assignments, eg, blue team, red team), and providers were educated on the workflow for forwarding results to nurses in the EMR.

Interrupted time series analysis was performed, whereby a bundle of interventions was rolled out, and the impact on outcome measures was analyzed.

### Data Analysis

To estimate the effect of our interventions, we created p charts for the 2–4 µg/dL and 5–9 µg/dL BLL ranges. These charts were created in R using a combination of ggplot2 and qicharts2. R is a programming language and environment for statistical computing.^18^ The upper control limit (UCL), lower control limit (LCL), and evidence of special cause variation were calculated using the Anhøj rules built into qicharts2. The Anhøj rules were chosen because they are more sensitive to the effect of sample size on control limits than traditional Institute for Healthcare Improvement or Six Sigma rules.^19,20^ According to the Anhøj rules, a shift is defined as:

Log2(n) + 3, where n is the sample size.

Our sample size (number of months analyzed) was 20. A shift is, therefore, defined as 7 points in a row above or below the centerline. The UCL and LCL were determined using Anhøj rules, and by normalizing our UCLs and LCLs to the preintervention period, we could detect any deviation outside that expected range.

**Fig. 4.**
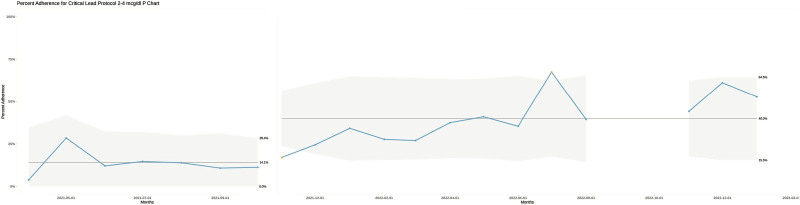
P chart showing the proportion of provider adherence to the critical lead protocol for blood levels 2–4 µg/dL over time between April 1, 2021, and February 1, 2023. The gray areas show the UCLs and LCLs of the process. The centerlines are solid black lines annotated with their values, 14.1% and 40%, respectively. A shift of >8 points above the centerline starts November 2021. Consequently, we split the graph at this point and calculated a new centerline at 40%, which is the mean provider adherence from November 2021 through the rest of the project. The shift, increase in the centerline, and subsequent distinct UCL and LCL ranges are evidence of special cause variance in the process. Individual points showing special cause variation are marked in yellow. During September–October 2022, there was a statewide contamination issue affecting the tubes used to collect pediatric lead samples, so data from these two months were excluded. Of note, this gap represents missing data, whereas the division of the graph before and after November 2021 does not represent missing data.

Consequently, the UCL and LCL for each month differ based on the sample size. Individual points exceeding the UCL or LCL are marked in yellow on our p charts, and the range between the UCL and LCL is gray. When a point exceeded the UCL to LCL range, we considered that evidence of special cause variation. In the case of Figure 4, we saw special cause variance starting in November 2021 and consequently split the p chart by creating a new centerline with new UCLs and LCLs for the remainder of the project time.

Additionally, postintervention mean provider adherence was calculated for the 2–4 and 5–9 µg/dL ranges, ≥10 µg/dL range, and globally (all ranges together) by calculating adherence for each range over the final 6 months of the study and then taking the mean of those values. This allowed a direct comparison with the preintervention period, which also spanned 6 months and was calculated similarly.

### IRB and Funding

The IRB evaluated this study and deemed it exempt, without ethical concerns. The researchers, who all worked at the BMC pediatric clinic during the study period, occasionally evaluated their own adherence rates during chart review. Still, we believe no or minimal bias was introduced as there were no rewards or penalties based on adherence rates. When repeated low adherence was noted, our team gave feedback to individual providers as per intervention (3) to ensure their patients received appropriate care. For all patients with a BLL of ≥10 µg/dL, part of the pathway was referring them to the BMC Lead Clinic, which offered specific follow-up. Additionally, for all BLLs ≥10 µg/dL, the Department of Public Health is automatically notified. It will reach out to providers if lead levels are not repeated within an appropriate time.

Funding from the BMC QI Grant was used to purchase stickers, pins, and magnets for the “2 is 2 High” campaign and to pay for access to data.

## RESULTS

We reviewed 1,166 charts and, ultimately 853 were included based on our eligibility criteria: 783 in the 2–4 µg/dL range, 58 in the 5–9 µg/dL range, and 12 in the ≥10 µg/dL range (Fig. 1). During September–October 2022, a statewide contamination issue affected the tubes used to collect pediatric lead samples. Data from these 2 months (291 charts) were excluded because there were many false-positive results, and providers had to deviate from the protocol by ordering repeat testing and altering their counseling in light of this issue. Those charts were not reviewed and are not counted in the 1,166 total. Postintervention means, as stated, include data from the last 6 months of the study, which included June, July, August, November, and December 2022, as well as January 2023, but excluded September and October 2022.

**Fig. 5.**
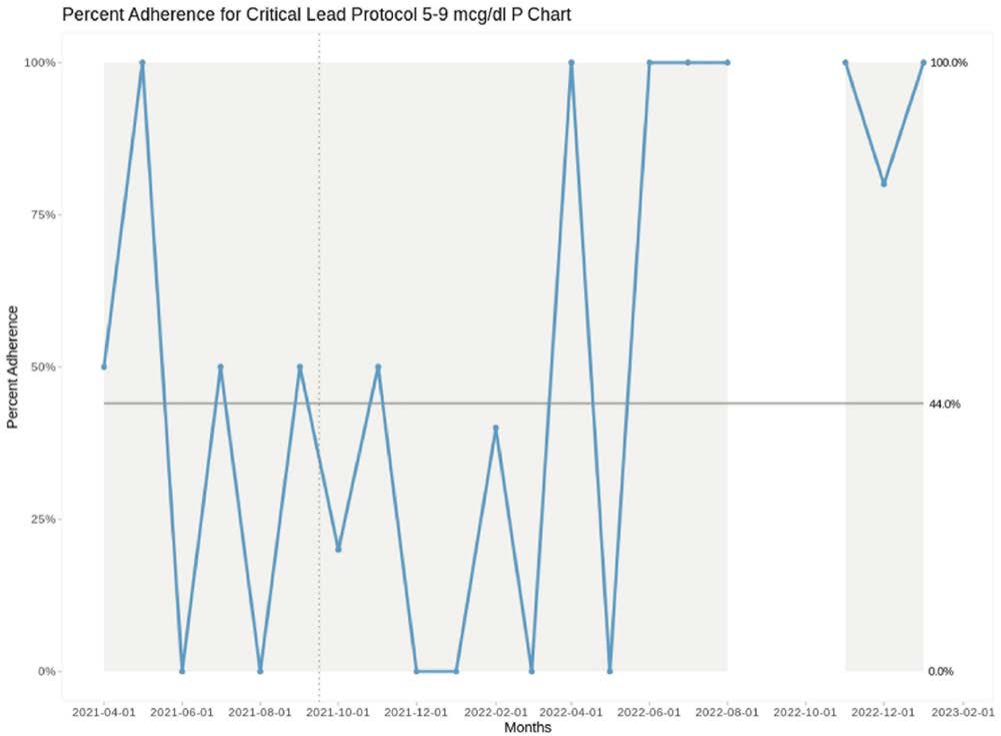
P chart showing proportion of provider adherence to the critical lead protocol for blood levels 5–9 µg/dL over time, between April 1, 2021, and February 1, 2023. The gray areas show the UCL and LCL of the process. The dashed vertical line represents the end of the preintervention period. During September–October 2022, a statewide contamination issue affected the tubes used to collect pediatric lead samples, so data from these 2 months were excluded.

In the 2–4 µg/dL group, mean provider adherence increased from 14.1% in the preintervention period to 50% in the final 6 months of the study, which we attribute to our interventions given the shift and special cause variation in the p chart for that BLL range (Fig. 4). In Figure 4, the centerline was recalculated starting in November 2021, marking the start of the period where special cause variation was observed. This new centerline of 40% represents the provider mean adherence from November 2021 through the end of the project. The new LCLs and UCLs around this centerline are also shown and are distinct from those in the preintervention range. In the 5–9 µg/dL group, the centerline remained 44% throughout, despite wide variation in monthly provider adherence (Fig. 5). The preintervention mean adherence for the 5–9 µg/dL group was 44% and the postintervention mean was 97%, but during the study period, there were no Statistical Process Control chart rules indicating special cause changes in protocol compliance. For the ≥10 µg/dL range, the preintervention mean was 0% and the postintervention mean was 75%, but there was insufficient data to analyze the effect of our interventions on these higher BLLs, as these events were rare (n = 12).

Global mean provider adherence increased to 53% measured across the final 6 months of the project, up from 16% in the 6 preintervention months. This change is largely driven by the improvement in the 2–4 µg/dL range which constituted the vast majority of the data (92%).

## DISCUSSION

This study demonstrated that simple COM-B framework-informed process changes could significantly impact adherence to a lead exposure protocol in a large, urban primary care practice. Using a series of multimodal interventions that improved providers’ capability, opportunity, and motivation to act on elevated BLLs, we changed provider behavior regarding following up on results in the 2–4 µg/dL range. Notably, the budget for this project was less than $1,000, and much of that funding was spent on data collection. Consequently, the interventions listed in this article are low cost and generalizable to any pediatric clinic that uses an EMR.

We believe the lack of change seen in the 5–9 µg/dL range is secondary to the small number of laboratory values in that range each month. This resulted in large fluctuations in adherence and a wide spread between the UCL and LCL. Only 6 points were documented above the centerline, which is 1 point shy of the definition of a shift using the underlying rule of our model and does not indicate a change. A more extended observation period and different data subsetting (possibly grouping the higher levels over multiple months rather than monthly) might make observing change in this group easier.

Although we demonstrated a change in the 2–4 µg/dL range, global adherence increased to only 53% at the end of this study, falling short of our aim of 80%. Although the exact reasons for this discrepancy are unknown, several possible contributing factors exist. First, it is difficult for providers to change long-held habits, such as treating a BLL of 5 µg/dL as the “actionable” cutoff for lead exposure. During our study period, BLLs less than 5 µg/dL did not flag as abnormal in the EMR, reinforcing incorrect beliefs. Additionally, our team defined adherence to the Lead Exposure Protocol quite stringently, meaning we may have missed some improvement in follow-up that did not meet our “critical protocol” criteria. We were also only able to measure adherence based on chart documentation. Anecdotally, we know that some providers changed their behavior but did not always document all the steps involved. Future studies could use qualitative interviews, surveys, or focus groups to evaluate provider experience of interventions and improve their efficacy.

Our study builds upon existing themes in the literature and provides new tools for study. As mentioned, Brown et al^16^ showed the importance of EMR-based interventions, individual clinician feedback or coaching, and the integration of nurse-directed patient education. In 2021, Davidson et al^21^ also showed that EMR-based interventions and provider feedback were key to changing provider behavior, though that study focused on lead screening rather than follow-up. Our study adds to the evidence supporting these interventions.

Our study expanded on the use of educational campaigns including eye-catching graphics, pins, stickers, magnets, and buttons to drive excitement about the lead protocol and motiviation to adhere to it. Such campaigns are low-cost and could be replicated by other groups aiming to increase awareness of and adherence to any protocol. To our knowledge, our study is also unique in its application of the COM-B framework to this issue, which other institutions can adapt and apply to their own contexts. Finally, our QI study was unique in describing a distinct protocol for BLLs ≥10 µg/dL. Therefore, a larger or longer QI study focusing on provider response to significantly elevated BLLs using our protocol would be a strong addition to the literature.

As with many QI projects, this project not only improved a process but also focused more institutional attention on that process. Because of this work, clinic leadership agreed to develop a nurse-driven follow-up program for pediatric primary care patients with BLLs between 2 and 9 µg/dL. Results within this range are automatically sent to a Nursing Lead Inbox (and ordering provider) via the EMR, and nurses follow up on these levels via telehealth, utilizing the SmartPhrases created for this project. This shows how a well-designed QI project can be used as a lever to shift institutional resources and priorities toward patient safety efforts that may otherwise be overlooked.

### Limitations

This study has several limitations. The number of children with elevated BLLs ≥10 µg/dL was small (n = 12), so it was difficult to assess the effectiveness of interventions for those ranges. To mitigate this, future studies could be extended to multiple centers or cover a longer time period. As mentioned, the data from 2 months of the study period (September and October 2022) were unusable due to statewide issues with contaminated collection tubes. However, our team has no reason to believe there would have been lasting impacts on the study after this period, as providers resumed normal protocol use in November of 2022. Third, no primary health outcome measures were tracked. Trending BLLs over time and assessing the correlation between protocol adherence and problems on a patient’s problem list, such as speech/language delay, could help provide more information about the health consequences of provider adherence, although data would be correlational. Finally, this study measured only what was documented in the chart. If providers did not document their follow-up steps, they were not counted as protocol-adherent.

### Equity and Next Steps

Lead exposures disproportionately impact lower-income communities and communities of color, making lead exposure a critical health equity issue. In Massachusetts in 2022, children living in low-income neighborhoods were nearly 3.6 times more likely to have elevated BLLs than children living in high-income communities; Black children were 1.6 times more likely than White children to have elevated BLLs, and children who identify as multi-race were 3.6 times more likely to have lead poisoning than White children.^22^ Future studies could stratify by geographic area to better characterize the patients experiencing these exposures at BMC. As BMC primarily serves patients from underserved areas of Boston and neighboring communities, many of whom speak a language other than English as their primary language or are new immigrants, we hope our interventions will improve outcomes related to the secondary effects of lead exposure in these groups. The next steps include translating the education section of our protocol and additional lead-related educational materials for families into a broader set of languages.

Simple and low-cost interventions increased adherence to the Lead Exposure Protocol at BMC’s Pediatric Primary Care Clinic. If other clinics have a standardized, evidence-based protocol for managing lead exposures or other routine screening results, these interventions could be adapted to improve adherence to those protocols.

## ACKNOWLEDGMENTS

The authors would like to thank the BMC QI Grant, in particular Dr. Spencer Wilson, Research Fellow in Quality and Patient Safety in the Department of Surgery at BMC, and Carrie Googins, Director of Quality and Patient Safety at BMC. This grant and support from Dr. Wilson and Ms. Googins allowed us to pay for access to the BMC CDW-R data each month and funded our “2 is 2 High” button/sticker/pin/poster educational campaign. Thank you to Erin M. Ashe, the Program Director of the BMC CDW-R, who helped us access chart review data each month. The authors also thank Chloe Rotman, Manager of Library Services at Boston Children’s Hospital, for helping with the literature review for this article. They would also thank Dr. Ryan Brewster for designing the “2 is 2 High” graphic.

## Supplementary Material


