# Supplementary material for: Improving Adherence to the Lead Exposure Protocol at Boston Medical Center’s Pediatric Clinic
Source: Pediatr Qual Saf. 2025 Feb 5;10(1):e793. doi: 10.1097/pq9.0000000000000793 (PMC11798393; doi:10.1097/pq9.0000000000000793)
Supplement: Supplementary file 1 [file pqs-10-e793-s001.pdf]

SDC Table 1: Timeline of Interventions

**Intervention**	**Category of Intervention**	**COM-B Tenet**	**Implementation Date**
Baseline Data Collection	N/A	N/A	April through September 2021
Lead Smart Phrase Introduction	EMR Support	Capability	September 2021
Resident and Preceptor Promotional Campaign	Promotion	Motivation	September to October 2021
Directed Feedback to Attending Providers	Feedback	Opportunity	April 2022 (continued during duration of the project)
Created Process for Nursing Team to Follow Up on Elevated Lead Levels 2 – 4 mcg/dl	Operational	Capability	May 2022
Modified Nursing Team Follow Up Process to be Simpler	Operational	Motivation	November 2022 (continued during duration of project)

SDC Table 1 Legend: Table of interventions and corresponding months in which they were introduced. This table also highlights the category and COM-B Tenet targeted by each intervention.
