# Supplementary material for: Improving Adherence to the Lead Exposure Protocol at Boston Medical Center’s Pediatric Clinic
Source: Pediatr Qual Saf. 2025 Feb 5;10(1):e793. doi: 10.1097/pq9.0000000000000793 (PMC11798393; doi:10.1097/pq9.0000000000000793)
Supplement: Supplementary file 2 [file pqs-10-e793-s002.pdf]

SDC Document 2 Legend: Examples of the full text/content of the SmartPhrases for BLL 2-4 mcg/dL and BLL 5-9 mcg/dL.

SmartPhrase: **.LEAD2TO4**

Text/Content of SmartPhrase:

RECOMMENDATIONS
1. Review lab results with family.
2. Take an environmental history and provide education on creating a lead safe environment.
3. Recheck lead level in 6-12 months. For children < 12 months, consider retesting in 3-6 months as lead exposure may increase as mobility increases.

ENVIRONMENTAL HISTORY (for sources of lead exposure)
1. Paint in homes or child care facilities built before 1978.
- Recent home renovations.
- Other locations where the child may spend time, such as daycare or relatives homes.
2. Soil contaminated by lead (near highways and in yard of houses with exterior lead paint).
3. Drinking water (household lead plumbing, lead soldered pipes)
4. Take-home exposures related to parental occupations (e.g. construction, auto repair, and plumbing).
5. Imported spices (e.g. Tumeric).
6. Cosmetics (e.g. Swad brand Sindoor powder, a cosmetic used in Hinduism; Kohl or Surma eye cosmetics from Africa, Middle East, or Asia).
7. Folk remedies (e.g. Greta and Azarcon, Hispanic traditional medicines; Ghasard, an Indian folk medicine; and Ba-baw-saw, a Chinese herbal remedy, all contain lead).
8. Old ceramic or antique cookware/imported cookware.
9. Toy jewelry.

EDUCATION (Home Lead Mitigation Strategies)
1. Keep surfaces clean with frequent wet mopping of floors, windowsills, and play areas.
2. Frequent handwashing with soap and water, especially before eating.
3. Cover areas of peeling or chipping paint with duct tape.
4. Remove shoes and change work clothes before entering the home, especially for those with high risk occupations (e.g. construction, auto repair, plumbing).
5. Run tap water to cold for 1-2 minutes prior to use.
6. Stop any ongoing renovations unless done by a lead certified contractor, taking precautions, such as not residing in home while interior renovations occur.
7. Home lead inspection by certified lead inspector or Department of Public Health worker.
- For lead levels >/= 10 mcg/dL, the city of Boston and state of Massachusetts is mandated to perform home lead inspection and abatement if lead is detected.
- For lead levels < 10 mcg/dL, families can hire a private inspector.

SmartPhrase: **.LEAD5TO9**

Text/Content of SmartPhrase:

RECOMMENDATIONS

1. Review lab results with family. Take an environmental history and provide education on creating a lead safe environment.
2. Add “elevated blood lead level” to the patient problem list.
3. Screen for iron deficiency with lab testing (CBC, ferritin, CRP) and provide iron supplementation if indicated.
4. Provide nutritional recommendations related to adequate iron, calcium, and vitamin C intake
5. Recheck lead level in 1-3 months to ensure it is not rising. If the lead level is stable or decreasing, retest in 3 months. Check lead level in siblings <6 years of age.

ENVIRONMENTAL HISTORY (for sources of lead exposure)
1. Paint in homes or child care facilities built before 1978.
- Recent home renovations.
- Other locations where the child may spend time, such as daycare or relatives homes.
2. Soil contaminated by lead (near highways and in yard of houses with exterior lead paint).
3. Drinking water (household lead plumbing, lead soldered pipes)
4. Take-home exposures related to parental occupations (e.g. construction, auto repair, and plumbing).
5. Imported spices (e.g. Tumeric).
6. Cosmetics (e.g. Swad brand Sindoor powder, a cosmetic used in Hinduism; Kohl or Surma eye cosmetics from Africa, Middle East, or Asia).
7. Folk remedies (e.g. Greta and Azarcon, Hispanic traditional medicines; Ghasard, an Indian folk medicine; and Ba-baw-saw, a Chinese herbal remedy, all contain lead).
8. Old ceramic or antique cookware/imported cookware.
9. Toy jewelry.

EDUCATION (Home Lead Mitigation Strategies)
1. Keep surfaces clean with frequent wet mopping of floors, windowsills, and play areas.
2. Frequent handwashing with soap and water, especially before eating.
3. Cover areas of peeling or chipping paint with duct tape.
4. Remove shoes and change work clothes before entering the home, especially for those with high risk occupations (e.g. construction, auto repair, plumbing).
5. Run tap water to cold for 1-2 minutes prior to use.
6. Stop any ongoing renovations unless done by a lead certified contractor, taking precautions, such as not residing in home while interior renovations occur.
7. Home lead inspection by certified lead inspector or Department of Public Health worker.
- For lead levels >/= 10 mcg/dL, the city of Boston and state of Massachusetts is mandated to perform home lead inspection and abatement if lead is detected.
- For lead levels < 10 mcg/dL, families can hire a private inspector.

NUTRITIONAL RECOMMENDATIONS
Iron: Encourage the consumption of iron-enriched foods (e.g. fortified cereals, meats, beans). Introduce pureed meats as soon as the child is developmentally ready. For older children, provide 1 serving of lean red meat per day. For iron deficiency, start iron supplementation with ferrous sulfate 3mg/kg/day. No indication for iron supplementation if not iron deficient.

Vitamin C: Iron absorption quadruples when taken with Vitamin-C containing foods. Recommend having a fruit at every meal to increase iron absorption. At least 2 servings per day of foods high in vitamin C (e.g. fruits, vegetables, or juice), preferably with meals to increase iron absorption.

Calcium: Recommend 2-3 servings per day of dairy products or other calcium-rich foods (e.g. milk, yogurt, and cheese).

RESOURCES FOR FAMILIES:

1. MA Department of Public Health: “Lead and Your Child’s Health”
   https://www.mass.gov/service-details/learn-about-short-term-habits-to-keep-your-child-safe-from-lead
2. US Environmental Protection Agency: “Lead and a Healthy Diet”
   https://www.epa.gov/sites/production/files/2020-01/documents/fight_lead_poisoning_with_a_healthy_diet_2019.pdf
3. Centers for Disease Control and Prevention, “Lead Factsheet”: https://www.cdc.gov/biomonitoring/Lead_FactSheet.html

Boston Public Health Commision contact information: (617) 534 - 5965

MA DPH Childhood Lead Poisoning Prevention Program contact information: (617) 624 – 5757
